# AVISPA: a web tool for the prediction and analysis of alternative splicing

**DOI:** 10.1186/gb-2013-14-10-r114

**Published:** 2013-10-24

**Authors:** Yoseph Barash, Jorge Vaquero-Garcia, Juan González-Vallinas, Hui Yuan Xiong, Weijun Gao, Leo J Lee, Brendan J Frey

**Affiliations:** 1 Department of Genetics, University of Pennsylvania, Philadelphia, PA 19104, USA; 2 Department of Computer and Information Science, University of Pennsylvania, Philadelphia, PA 19104, USA; 3 Universitat Pompeu Fabra, Barcelona 08003, Spain; 4 Department of Electrical and Computer Engineering, University of Toronto, Toronto, ON M5S 3G4, Canada; 5 Banting and Best Department of Medical Research, University of Toronto, Toronto, ON M5G 1L6, Canada

## Abstract

Transcriptome complexity and its relation to numerous diseases underpins the need to predict *in silico* splice variants and the regulatory elements that affect them. Building upon our recently described splicing code, we developed AVISPA, a Galaxy-based web tool for splicing prediction and analysis. Given an exon and its proximal sequence, the tool predicts whether the exon is alternatively spliced, displays tissue-dependent splicing patterns, and whether it has associated regulatory elements. We assess AVISPA's accuracy on an independent dataset of tissue-dependent exons, and illustrate how the tool can be applied to analyze a gene of interest. AVISPA is available at http://avispa.biociphers.org.

## 

Alternative splicing (AS) is estimated to affect transcripts from over 95% of human multi-exon genes [1,2], with the most common class of AS involving cassette exons. Thousands of alternative cassette exons have been found to be differentially spliced between mammalian tissues, with tissues such as the brain displaying the most complex patterns [1,2]. These observations and the association of many splicing defects with diseases [3] motivated the recent derivation of a splicing code. The code, comprising a model with a set of rules that can predict splicing outcomes given genomic sequence and cellular context [4,5], used over 1,000 regulatory features. Trained using inclusion measurements for 3,700 cassette exons across 27 mouse tissues, the code’s model was shown to predict differential AS in four tissue groups: the central nervous system (CNS), muscle, digestive, and embryo versus adult tissues.

The derivation of a predictive splicing code served as proof-of-concept and enabled insights into RNA biogenesis [5,6], but was limited in scope. Specifically, it was only applied to a subset of alternative exons in specific studies. However, given the importance of splicing in the study of gene regulation, development and disease, it became important to translate the splicing code models into a tool that would be accessible for researchers in a wide range of fields. Here, we present AVISPA (Advanced Visualization of Splicing Prediction and Analysis), a web tool that enables both prediction and splicing analysis of alternative and tissue-dependent exons in any gene of interest. Given an exon, the tool predicts whether it is alternative and whether its inclusion is expected to change in different tissues. It reports whether the exon is known to be alternative based on an internal transcripts database, and performs *in silico* splicing analysis, identifying putative regulatory elements and mapping those as tracks in the genome browser.

AVISPA’s pipeline is illustrated in Figure 1. Users submit a query by specifying the sequence or genomic coordinates of either a single exon, or a triplet of exons that includes the immediate up- and downstream exons of the query exon. In the pre-processing step, the query is matched against an internal database of exon triplets mined from known transcripts and mapped to the reference genome. The result of the pre-processing is reported in the AVISPA’s output and indicates existing evidence for whether the exon is alternatively spliced based on, for example, alignments of cDNA and EST data. After the query has been successfully matched, RNA features are extracted from the query exon and flanking regions [5]. At the first prediction stage, the extracted features are used to predict whether the query exon is alternatively or constitutively spliced. If the query is predicted to be an alternative cassette exon, a second prediction step assesses whether the exon is differentially included in specific tissues.

**Figure 1:**
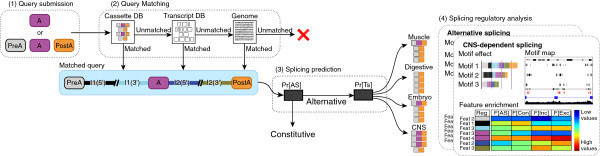
**AVISPA’s analysis pipeline.** The analysis is composed of the following steps. (1) Query submission: users submit a query composed of either a single exon of interest or an exon triplet that also specifies the up- and downstream exons. (2) Query matching: the submitted query is first matched against internal databases (DB) of known transcripts and alternative exons. If no match is found the query is searched against the reference genome. If the query cannot be matched (red cross) an error is reported. (3) Splicing prediction: a successfully matched query (light blue rectangle) is scored as an alternative cassette exon, followed by scoring for differential splicing in four tissue groups. (4) Splicing analysis: if the query’s predictions pass a user-defined significance threshold a splicing analysis is performed. Analysis includes feature enrichment, effect of *in silico* motif removal on splicing predictions, and mapping putative regulatory motifs to the genome. A visual summary of both predictions and splicing analysis is produced (right).

The new web tool offers marked improvements over available software. First, it offers 'genome-wide' tissue-dependent splicing predictions, where any exon can be submitted as a query. By contrast, the original work only allowed analysis on a previously mined set of approximately 12,000 cassette exons, while other tools focus on quantifying experimental data or general splice site and motif analysis [7-9]. Second, AVISPA offers a new *in silico* analysis of regulatory features and the mapping of putative regulatory sequence motifs in the genome. As part of this analysis, motifs found to be robustly included in the Bayesian ensemble of models and present in the query are removed *in silico* to determine their effect on splicing prediction. The relative effect of these feature removals is reported as a bar chart of the normalized feature effect (NFE). The putative regulatory motifs are also mapped to the genome using the UCSC genome browser, where they can be combined with other tracks, such as known single nucleotide polymorphisms and binding measurements of known splicing factors [10]. Additionally, the enrichment of the query’s features is compared to reference groups such as alternative or constitutively spliced exons in AVISPA’s database. Feature enrichment is reported using a standard heat map ranging from blue, for relatively low values, to red for relatively high values. For example, a relatively strong 3’ splice site will appear red, indicating a high score, while a weak splice site will be marked blue.

The new tool also includes several other improvements. First, the prediction technique is now based on a Bayesian neural network, which provides improved prediction accuracy compared to a battery of other methods [11]. Second, the original dataset of 3,700 cassette exons has been expanded to approximately 30,000 exons using data from 33 experiments in 11 mouse tissues [12]. Third, AVISPA uses an extended set of features that include computationally predicted nucleosome occupancy [13] together with primary sequence motifs implicated in general splicing regulation.

## Assessing splicing prediction accuracy

**Figure 2:**
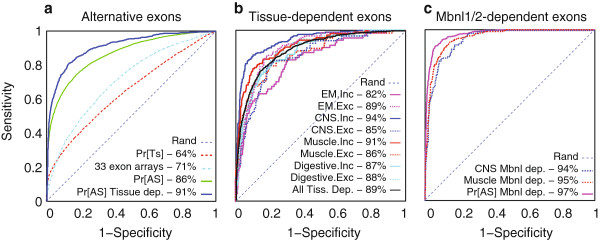
**Prediction accuracy. (a)** Differentiating alternative (n = 11,773) from constitutive (n = 9,638) exons. Detecting which exons are alternative (green) is significantly improved compared to a classifier that uses exon expression measurements from 33 experiments (cyan), and compared to the original classifier trained to detect only tissue-dependent cassette exons (red). Detection of exons that exhibit tissue-dependent splicing changes (blue, n = 659) is much more accurate. Numbers within each legend represent the area under the curve (AUC) **(b)** Identifying tissue-dependent splicing. Detecting tissue-dependent splicing changes (n = 865) from a random set of non-tissue-dependent exons (n = 4,000) achieves an overall accuracy of 89% AUC (black). Accuracy varies considerably between tissues and for detecting increased inclusion (solid line) or exclusion (dashed) in a tissue **(c)** Detection accuracy for an independent set of *Mbnl*1/2-dependent exons [14] (n = 461). Differentiating between *Mbnl*1/2-dependent exons and constitutive exons achieves 97% AUC. Accuracy in detecting *Mbnl*1/2-dependent exons from a random set of non-tissue-dependent exons (n = 2,000) is approximately 94% AUC for both brain (blue) and muscle (red).

The new two-stage prediction paradigm, combined with the expanded dataset, yields a significant improvement in detecting alternative cassette exons (Figure 2a). For example, using only tissue-dependent splicing predictors achieves an area under the curve (AUC) of 64% for distinguishing between alternative and constitutive exons, compared to 86% by the first stage classifier. The improved accuracy of 94% AUC achieved for detecting tissue-dependent exons is to be expected, as many regulatory features and higher intronic conservation are associated with such exons. Notably, AVISPA’s sequence-based predictions offer a significant improvement compared to a similar classifier that directly uses normalized exon expression measurements from 33 experiments [12]. The latter achieves an overall lower accuracy of 71% AUC, with a significantly 2.5-fold lower sensitivity (54% versus 21%) for high-confidence events at a false positive rate of 2%. These results illustrate the usefulness of the new tool, which generalizes over experimental conditions and is not limited by technical factors such as microarray noise or read coverage. We note that these accuracy estimates can be considered as lower bounds, as some of the events labeled as constitutive in our database may be alternative.

The new tool also achieves significant improvement in detecting tissue-dependent exons (Figure 2b). The overall accuracy in discriminating between tissue-dependent and non-tissue-dependent exons is 89% AUC, but varies considerably between tissues and between differential inclusion and exclusion in the same tissue type. For example, the highest accuracy was achieved for detecting increased inclusion of exons in CNS (94% AUC) and muscle tissues (91% AUC), while the lowest accuracy was for detecting increased exclusion in CNS (85% AUC) and increased inclusion in embryonic tissues (82% AUC).

In order to test AVISPA on an independent dataset, we computed predictions for a set of cassette exons recently shown to be regulated by the Muscleblind-like proteins *Mbnl*1/2 in mouse brain, muscle, and heart [14]. Figure 2c shows AVISPA easily distinguished these exons from constitutive exons (97% AUC), similar to its performance in detecting tissue-dependent alternative exons in the original test set. In discriminating the Mbnl1/2-regulated exons from non-CNS- and non-muscle-dependent exons, AVISPA achieves an AUC of 93% and 94%, respectively, while *in silico* removal of *Mbnl*1/2 caused, on average, an almost two-fold larger effect for *Mbnl*1/2-regulated exons compared to the effect for non-muscle- and non-heart-dependent exons. The improved accuracy in detecting *Mbnl*1/2-regulated exons compared to the detection of tissue-dependent exons in the original test data is likely due to a lower false detection rate from the RNA-Seq and CLIP-Seq experiments in [14].

Finally, we also tested whether the regulatory features added in the web tool were useful for splicing prediction. As expected, many of the sequence motifs implicated in general splicing regulation were included in the code, especially for differentiating between alternative and constitutive exons. By contrast, the relation between nucleosome occupancy and alternative splicing is less well understood, and has garnered much research attention [15,16]. We found that the model selected features representing nucleosome occupancy around the alternative exon, but training the model without these features resulted in similar prediction accuracy (data not shown). This result indicates that other features in our model, such as di- and tri-nucleotide frequencies, already captured the 'predictive power' of computationally derived nucleosome position features.

### *Vegfa in silico* splicing analysis

Previous work demonstrated how the splicing code model could be used to identify new regulatory elements, detect novel tissue-dependent splicing events, and study the evolution of splicing across vertebrates [6]. Here, we illustrate how the new tool can be used to analyze a well-studied gene of major interest. We applied AVISPA to the vascular endothelial growth factor A (*Vegfa*) gene. *Vegfa* has a complex and highly conserved pattern of alternative splicing that changes across tissues and developmental stages [17,18]. Its role in angiogenesis, which is controlled in part by alternative splicing, has made it an attractive target of several anticancer therapies. Accordingly, there is considerable interest in identifying the factors that regulate the splicing of *Vegfa* transcripts [18,19]. Analyzing all *Vegfa* exon triplets revealed that only exons 6 and 7 were predicted to be cassette exons, with a score corresponding to a false positive rate of 0.009 and 0.017, respectively. For comparison, other exons scores corresponded to a false positive rate of 0.22 or higher (data not shown). These predictions are in line with annotated transcripts, many of which skip exon 6, one that skips exon 7 (ENSMUST00000113519), and several that skip both. Exons 6 and 7 were also both predicted, with a false positive rate of less than 0.025, to exhibit differential splicing in all four major tissue groups modeled. While confidence in differential splicing was high, the predictions were not conclusive as to whether a relative increase or decrease of exon inclusion would occur in the tissues. These results reflect the conserved and complex splicing pattern of *Vegfa*, with RT-PCR experiments showing exon 6 to have a complex bi-phasic increase of inclusion in developing mouse and chicken heart [18]. Prediction of other splice variations of *Vegfa*, such as the 3’ splice site variation in exon 8, are currently not supported by the tool.

**Figure 3:**
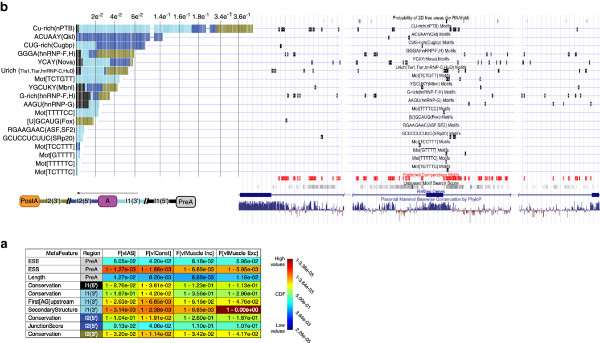
**Analysis of *Vegfa *exon 6 muscle-dependent inclusion.** A subset of the summary page produced by AVISPA is shown. **(a)** Feature enrichment analysis: the values of the features listed on the left are computed for *Vegfa* exon 6 and compared against matching feature values in a set of labeled exons. The four sets of exons compared against here are alternative exons ('AS', third column from the left), constitutive exons ('Const', third column from the right), exons differentially included in muscle ('Muscle Inc', second column from the right), and differentially excluded in muscle ('Muscle Exc', right most column). Relative enrichment or depletion of features is indicated using the heat map on the right. Only features with significantly low (blue) and high (red) values are shown here. The genomic region of each feature is indicated by the second from left column using the notation and colors in the top figure. **(b)** Stacked bar chart (left) of the normalized feature effect (NFE, y-axis) on splicing prediction. Only the top motifs are shown. Motif regions are annotated using the color scheme depicted below. Mapping of the motifs onto the UCSC genome browser is shown on the right. Tracks combining all motifs used by the code model (red), the unbiased motif search [5] (grey scaled), and conservation (blue) are added at the bottom.

Figure 3 shows the regulatory feature analysis for differential inclusion of *Vegfa* exon 6 in muscle. The enrichment analysis in Figure 3a highlights that the alternative exon is depleted of non-tissue-specific exonic splicing enhancers and is highly enriched with exonic splicing silencers. Other highlighted features are enriched secondary structure-free regions in the upstream intron, a distant first AG nucleotide upstream and a particularly short preceding exon 5. The preceding exon, for example, is 32 bp long, and the enrichment analysis indicates that only 0.127% of the tool’s reference set of alternative exons has a shorter preceding exon. The most dominant effect of *in silico* motif removal (Figure 3b) is for CU-rich elements known to bind Ptb1/2, followed by an ACUAAY motif known to bind *Quaking* (Qk). These splice factors have not been previously reported to regulate *Vegfa*, but a recent study estimates 39% of regulated exons during myogenesis are under the control of one or both of these splicing factors [20]. A smaller effect on splicing prediction in muscle is associated with intronic motifs known to bind Cugbp1/2 and Muscleblind-like protein (*Mbnl*1/2). Both Cugbp1/2 and *Mbnl*1/2 have been shown to play an important role in regulating splicing in developing hearts. Overexpressing *Cugbp1* or knockdown of *Mbnl1* in the adult mouse heart did not alter exon 6 inclusion levels significantly [18], but recent results point to possible compensatory effects between *Mbnl1* and *Mbnl2*[14]. Other elements implicated in *Vegfa* splicing regulation include the short YCAY motifs known to bind Nova proteins [21] and a UGCAUG motif, known to bind the brain- and muscle-specific splicing factor *Fox-1* (*A2bp1*) and its paralog *Fox-2* (*Rbm9*) [22]. While the *Fox-1/2* binding site is highly conserved, it resides over 1 kb downstream of exon 6 and *Fox-1/2* have not been previously reported to regulate *Vegfa.* However, recent results indicate that *Fox-2* knockdown in mice clearly alters *Vegfa* splicing pattern during heart development (Xiang-Dong Fu, personal communication). Smaller effects associated with non-tissue-specific regulation include G-rich elements, known to bind *hnRNP-F/H*, and U-rich elements that are known to bind *hnRNP-C* and *Tiar/Tia1*[23]. Notably, *Tia1* was previously reported to regulate *Vegfa* isoform expression [24]. Overall, our exploratory analysis of *Vegfa* splicing is consistent with previous results and offers new insights into mechanism of *Vegfa* regulation that are supported by recent experiments.

In summary, we presented a new tool, AVISPA, for *in silico* prediction and analysis of alternative splicing. The tool is not limited by technical constraints such as sequencing depth, and its predictions for alternatively spliced exons generalize over unmeasured conditions. Beyond the splicing outcome, it offers researchers the ability to identify putative regulatory elements and map those to the genome. These capabilities were recently used in an independent study to identify *TIA1* as a regulator of an alternative exon coding *miR-412*[25]. Here, we used a recent genome-wide study to demonstrate the tool’s accuracy for predicting muscle, heart, and brain regulated exons and performed detailed *in silico* splicing analysis for the vascular endothelial growth factor A.

Several important elements remain as on-going and future enhancements of the tool. These include predictions for species other than mouse, predictions for additional forms of alternative splicing (for example, alternative 3’ and 5’ splice sites), and higher resolution of tissue specificity. Currently, AVISPA’s predictions reflect confidence in alternative splicing or in relative, tissue-dependent, inclusion changes. Thus, users may infer an exon is likely to be alternative or to be differentially included in brain versus other tissues, but predictions for absolute inclusion levels (for example, 20% inclusion in brain, 40% inclusion in liver) are currently not supported. The tool has some technical limitations as well. Users can only submit a single cassette exon as a query, due to the computational burden involved in processing a query. Queries must be based on annotated exons, cannot contain exons shorter than 10 bases long, and non-canonical splicing by the minor spliceosome is not supported. Nonetheless, the ability to perform splicing prediction irrespective of experimental limitations, coupled with the new regulatory elements analysis, should serve researchers studying gene regulation, RNA biogenesis, and development. Moreover, AVISPA is built as a flexible platform that can be repeatedly updated as more data and improved models become available. The new computational analysis offered by AVISPA should facilitate the discovery of novel splicing variants, regulatory elements, and genomic variations affecting phenotypic variability or disease.

## Materials and methods

### Query matching against sequence database

The web-tool’s internal database includes three components. The first is a database of 11,773 cassette exons that we previously mined from sequence libraries [5]. The second is a set of 9,638 exon triplets derived from Refseq [26] and other sequence libraries as described in [5], where every three constitutive exons in a transcript define a triplet. These triplets were also scanned against exon expression measurements in 11 mouse tissues [12] and triplets suspected to contain an alternative cassette exon were removed. A query’s sequence is matched against the two transcript databases using BLAT with parameters set to tileSize = 8, minMatch = 2, minIdentity = 88. The third database component is the mouse assembly mm10 from the UCSC Genome Browser [27]. Matching a query to the reference genome is executed only if no match in the two transcript-based databases is found, and only when genomic coordinates for all three exons are specified.

### Extended regulatory feature set

We extended the set of putative regulatory features to include the occurrences of 350 new binding motifs in the seven regions around a cassette exon as defined in [5]. The motifs correspond to general splicing related RNA binding proteins (RBPs), SR and SR-related proteins (SC35, SRp20, 9G8, ASF/SF2, SRp30c, SRp38, SRp40, SRp55, SRp75, Tra2α/β), and hnRNP proteins (hnRNPA1, hnRNPA2/B1, hnRNPF/H, hnRNPG).

We also added features encoding computationally predicted nucleosome occupancy around the alternative exon [13]. Features were defined as the average and maximal occupancy scores in the first 100 nucleotides in each intron and the first or last 50 nucleotides of the alternative exon.

### Extended training set for tissue-specific alternative splicing

A total of 33 data tracks for normalized expression measurements using Affymetrix exon arrays were downloaded from the UCSC Genome Browser. The tracks are composed of measurements in 11 mouse tissues (brain, embryo, heart, kidney, liver, lung, muscle, ovary, spleen, testis, thymus) with three replicates for each tissue [12]. The expression of each exon and the relative inclusion of a putative cassette exon compared to its flanking exons were used as input features to train an ensemble of Bayesian neural networks [11]. The networks used these input features to identify differential inclusion and exclusion of alternative exons in the four tissue groups previously identified (CNS, muscle, digestive, embryo). Training was based on a subset of 3,770 cassette exons for which three probabilities for increased inclusion (*q*^inc^), increased exclusion (*q*^exc^) and no change (*q*^nc^) in each of the four tissue groups was previously computed [5]. This training step allowed the calibration of differential splicing estimation obtained from the new set of 33 experiments to the estimates used to train the original splicing model [5]. The model ensemble was then used to estimate differential splicing (*q*^inc^,*q*^exc^,*q*^nc^) for the remaining exons. The differential splicing estimates for the original set of 3,770 exons were averaged between the two datasets and care was taken to make sure predictions were based on non-overlapping training sets.

### Predicting alternative cassette exons using expression data and a single stage tissue-specific classifier

The 33 expression data tracks described above were also used to train a Bayesian deep neural network classifier [11], denoted '*33 exon arrays*' in Figure 2a. Any exon triplets from the set of 11,773 cassette exons and 9,638 putative constitutive exons that had missing data were removed, maintaining a total of 8,986 for training and test purposes.

The prediction of alternative exons using a single stage tissue classifier, denoted *Pr[Ts]* in Figure 2a, used a max function over the chance of differential splicing (1 - *p*^nc^) in each tissue.

### Training a splicing code model for alternative exons and for tissue-dependent splicing

For the purpose of inferring a regulatory model, we used a Bayesian neural network that worked better for this task than support vector machines, boosted decision trees, and other leading machine learning techniques [11]. To discriminate between alternative and constitutive exons the network was set to have 10 hidden units and a sparsity prior of 0.9 for connections between features and hidden units. For predicting tissue-dependent splicing the network was set to have 20 units and a sparsity prior of 0.95. Varying the sparsity prior between 0.85 and 0.95 and adding up to 10 more hidden units did not have a significant effect on the results (data not shown). An ensemble of 5,000 models generated by Markov chain Monte Carlo simulations was used to estimate differential splicing (*q*^inc^,*q*^exc^,*q*^nc^) as was previously described [11].

### Scoring tissue-dependent splicing

Under the new framework the probability that any given triplet of exons contain a tissue-dependent cassette exon can be expressed as:

$$P\left(O^{t}=\mathrm{ch}|r_{e}\right)=P\left(\mathrm{AS}|r_{e}\right)P\left(O^{t}=\mathrm{ch}|r_{e},\mathrm{AS}\right),$$

where *P*(*O*^*t*^ = *ch*|*r*_*e*_) denotes the probability to observe a change in the exon’s inclusion level in tissue *t* given the exon’s feature vector *r*_*e*_, *P*(*AS*|*r*_*e*_) is the probability the exon is alternative, and *P*(*O*^*t*^ = *ch*|*r*_*e*_, *AS*) is the probability of observing differential splicing given that the exon is alternative. The first term on the right is computed by the first stage predictor, while the second term is computed by the second stage predictor.

### ROC performance evaluation

Receiver operating characteristic (ROC) performance was evaluated using repeated five-fold cross-validation and care was taken to make sure predictions were based on non-redundant training sets, as was previously described [5]. Evaluation of discriminating between alternative and constitutive exons was based on a set of 11,773 cassette exons and 9,638 putative constitutive exons derived from EST/cDNA sequences [5]. In order to assess the accuracy of detecting cassette exons that exhibit a tissue-dependent splicing pattern (*for example,* differential inclusion in muscle) we compared the scores of such exons to those of a random set of exon triplets that do not exhibit this splicing pattern. The random set was selected using the following procedure. First, we used the 33 genome-wide exon expression measurements described above to quantify the inclusion level of all exon triplets from all Refseq transcripts. Next, we discarded triplets with missing data and required the relative expression of the upstream and downstream exons to be no more than 1.5-fold apart in all experiments. In order to avoid probe sets with little signal, we required the up- and downstream exons to have a normalized absolute value of at least 0.1 in at least 15 experiments. Additionally, we required in at least three experiments of the tissue group of interest (*for example,* digestive) that the up- and downstream exons are not in the bottom 20 percentile. Finally, the relative expression of each middle exon compared to its flanking exons was used to estimate the chance it is differentially included in each tissue group [28]. Any triplet that had a *P*-value of 0.7 or higher was deemed non-tissue-dependent and a set of approximately 2,000 exons was then selected for each tissue as a non-tissue-dependent exon set. Exons were selected randomly from the respective genes and then randomly from the relative order within the gene. We then verified that these are not biased in terms of relative location within the gene or gene length compared to a random sample of triplets from the genome (data not shown).

While small variations in the parameters of the above process did not have a notable effect on the results, we did detect an apparent selection bias in this procedure. Specifically, using expression measurements to select exons based on high confidence in non-tissue-dependent splicing may favor constitutive exons. Notably, the 'true' labels of any given exon as alternative or constitutive is unavailable. However, since our prediction algorithm has proved accurate in distinguishing alternative from constitutive exons (Figure 2a), we applied it to the set of 2,000 non-tissue-dependent exons selected for each tissue group. Compared to a random set of 1,000 exon triplets, these exons were biased towards constitutive exon scores (Additional file 1). To correct for this apparent bias we subsampled 1,000 exons for each tissue group so that their scores as alternative match those in the random set (Additional file 1, green and red lines). This corrected set of a total of 4,000 predictions was then used for subsequent analysis (Figure 2b,c). We note that without this correction the initial set of non-tissue-dependent exons results in improved performance compared to that shown in Figure 2.

### *In silico* feature removal and normalized feature effect

In order to evaluate the relative effect of a putative regulatory sequence motif (for example, the occurrence of a [U]GCAUG motif, known to bind *Fox*1/2, upstream of the alternative exon), the feature is first set to zero. The splicing predictions with the mutated feature, denoted $\left(p_{∆f}^{\mathrm{inc}},p_{∆f}^{\mathrm{exc}}\right),$ are then computed with the total effect on differential splicing defined as $\mathrm{FE}{}_{f}=|p^{\mathrm{inc}}-p_{∆}^{\mathrm{inc}}|+|p^{\mathrm{exc}}-p_{∆}^{\mathrm{exc}}|$. This definition aims to capture the effect of features that not only change the confidence in a splicing change $\left(p^{\mathrm{nc}}-p_{∆f}^{\mathrm{nc}}\right),$ but also change the relative confidence in either differential inclusion or exclusion. Finally, the normalized feature effect (NFE) is defined as:

$$\mathrm{NF}E_{f}=\frac{FE_{f}}{\sum_{j\in J}FE_{j}}$$

where *J* is the set of robust features. By itself, the NFE has no statistical significance measure associated with it. The NFE serves mainly as a quantitative tool to guide researchers interested in knowing which of the identified regulatory features have a higher effect on the model’s prediction confidence.

## Abbreviations

AS: Alternative splicing; AUC: Area under the curve; AVISPA: Advanced visualization of splicing prediction and analysis; CNS: Central nervous system; EST: Expressed sequence tag; NFE: Normalized feature effect.

## Competing interests

The authors declare that they have no competing interests.

## Authors’ contributions

YB and BJF conceived of the project. YB developed the combined prediction framework and *in silico* feature analysis. YB, JVG and WG developed the analysis pipeline with input from all authors. YB, JVG, WG and LJL created the sequence databases. JGV, WG and JVG developed the web tool. HYX, YB and BJF developed the prediction algorithms. YB and JVG performed the data analysis. YB wrote the paper with input from BJF. All authors read and approved the final manuscript.

## Supplementary Material

Additional file 1: Figure S1 Correcting constitutive exons selection bias in non-tissue-dependent exons. Exon scores for being alternative versus constitutive (x-axis) are plotted as a cumulative distribution function (CDF, y-axis). The initial set of selected non-tissue-dependent exons (blue) was biased towards constitutive exons compared to a random sample of 1,000 exon triplets from the genome (red). Subsampling the original set of 2,000 exons per tissue to fit the score distribution of a random set gave a good fit (green). Both green and red line plots are accumulated over all exons in all tissues as no significant difference was observed between the different tissues. Click here for file
