# Supplementary figures and images for: AVISPA: a web tool for the prediction and analysis of alternative splicing
Source: Genome Biol. 2013 Oct 24;14(10):R114. doi: 10.1186/gb-2013-14-10-r114 (PMC4014802; doi:10.1186/gb-2013-14-10-r114)

Exon scores as alternative cassette events

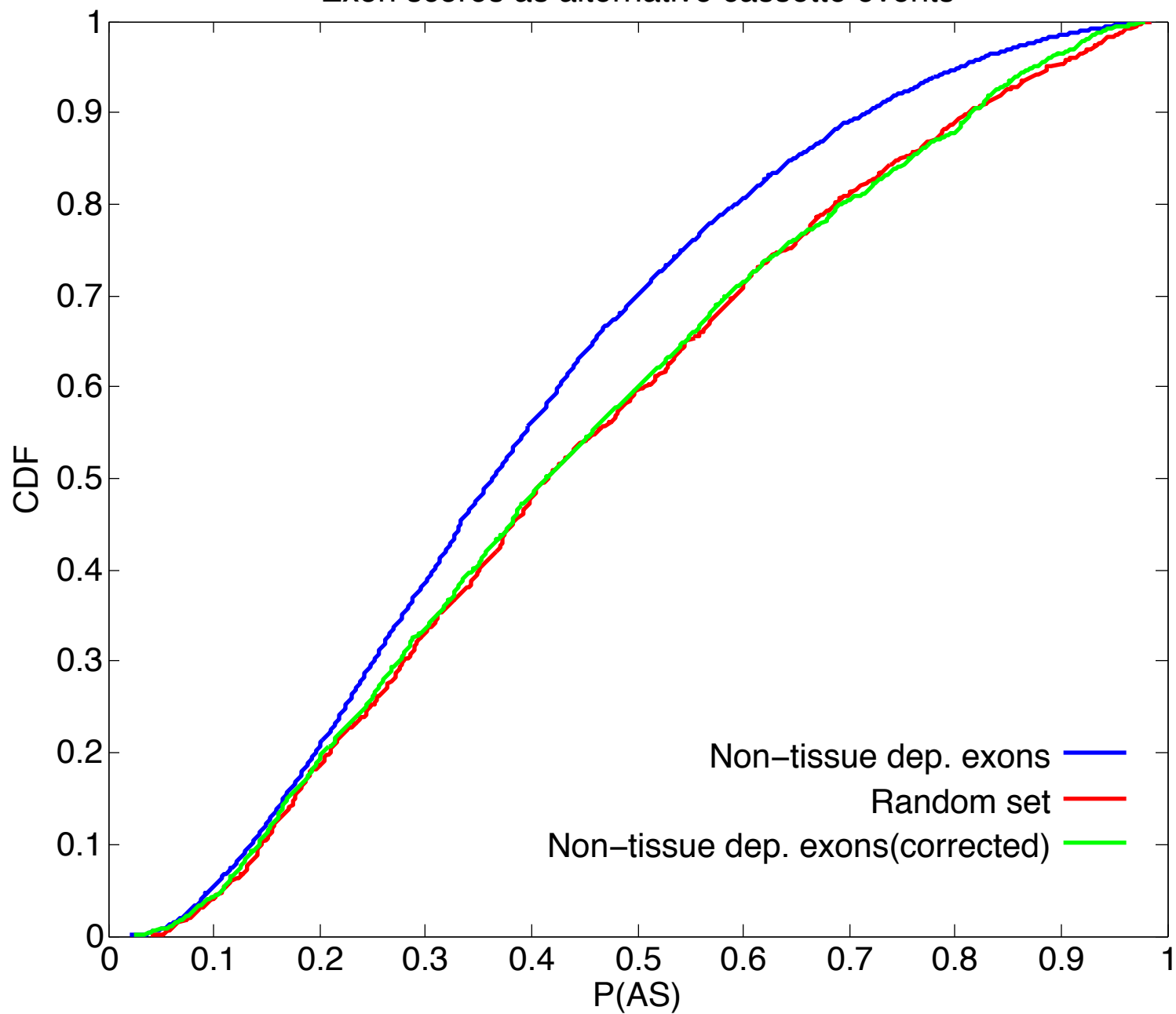

Supplement: Additional file 1: Figure S1 — Correcting constitutive exons selection bias in non-tissue-dependent exons. Exon scores for being alternative versus constitutive (x-axis) are plotted as a cumulative distribution function (CDF, y-axis). The initial set of selected non-tissue-dependent exons (blue) was biased towards constitutive exons compared to a random sample of 1,000 exon triplets from the genome (red). Subsampling the original set of 2,000 exons per tissue to fit the score distribution of a random set gave a good fit (green). Both green and red line plots are accumulated over all exons in all tissues as no significant difference was observed between the different tissues. [file gb-2013-14-10-r114-S1.pdf]
